# Relative free-energy calculations for scaffold hopping-type transformations with an automated RE-EDS sampling procedure

**DOI:** 10.1007/s10822-021-00436-z

**Published:** 2022-01-03

**Authors:** Benjamin Ries, Karl Normak, R. Gregor Weiß, Salomé Rieder, Emília P. Barros, Candide Champion, Gerhard König, Sereina Riniker

**Affiliations:** Laboratory of Physical Chemistry, ETH Zürich, Vladimir-Prelog-Weg 2, 8093 Zürich, Switzerland

**Keywords:** Molecular dynamics, Free energy calculation, Protein-ligand binding, Replica exchange, Enveloping distribution sampling

## Abstract

The calculation of relative free-energy differences between different compounds plays an important role in drug design to identify potent binders for a given protein target. Most rigorous methods based on molecular dynamics simulations estimate the free-energy difference between pairs of ligands. Thus, the comparison of multiple ligands requires the construction of a “state graph”, in which the compounds are connected by alchemical transformations. The computational cost can be optimized by reducing the state graph to a minimal set of transformations. However, this may require individual adaptation of the sampling strategy if a transformation process does not converge in a given simulation time. In contrast, path-free methods like replica-exchange enveloping distribution sampling (RE-EDS) allow the sampling of multiple states within a single simulation without the pre-definition of alchemical transition paths. To optimize sampling and convergence, a set of RE-EDS parameters needs to be estimated in a pre-processing step. Here, we present an automated procedure for this step that determines all required parameters, improving the robustness and ease of use of the methodology. To illustrate the performance, the relative binding free energies are calculated for a series of checkpoint kinase 1 inhibitors containing challenging transformations in ring size, opening/closing, and extension, which reflect changes observed in scaffold hopping. The simulation of such transformations with RE-EDS can be conducted with conventional force fields and, in particular, without soft bond-stretching terms.

**Supplementary Information:**

The online version contains supplementary material available at 10.1007/s10822-021-00436-z.

## Introduction

Rigorous free-energy calculations using molecular dynamics (MD) simulations have become an important tool to estimate binding free energies of novel compounds for lead optimization in drug discovery [1–3]. Although computationally relatively expensive, these methods are needed to properly account for entropic contributions introduced by protein/ligand conformational changes, entropy–enthalpy compensation, and desolvation of the ligand [4].

**Fig. 1:**
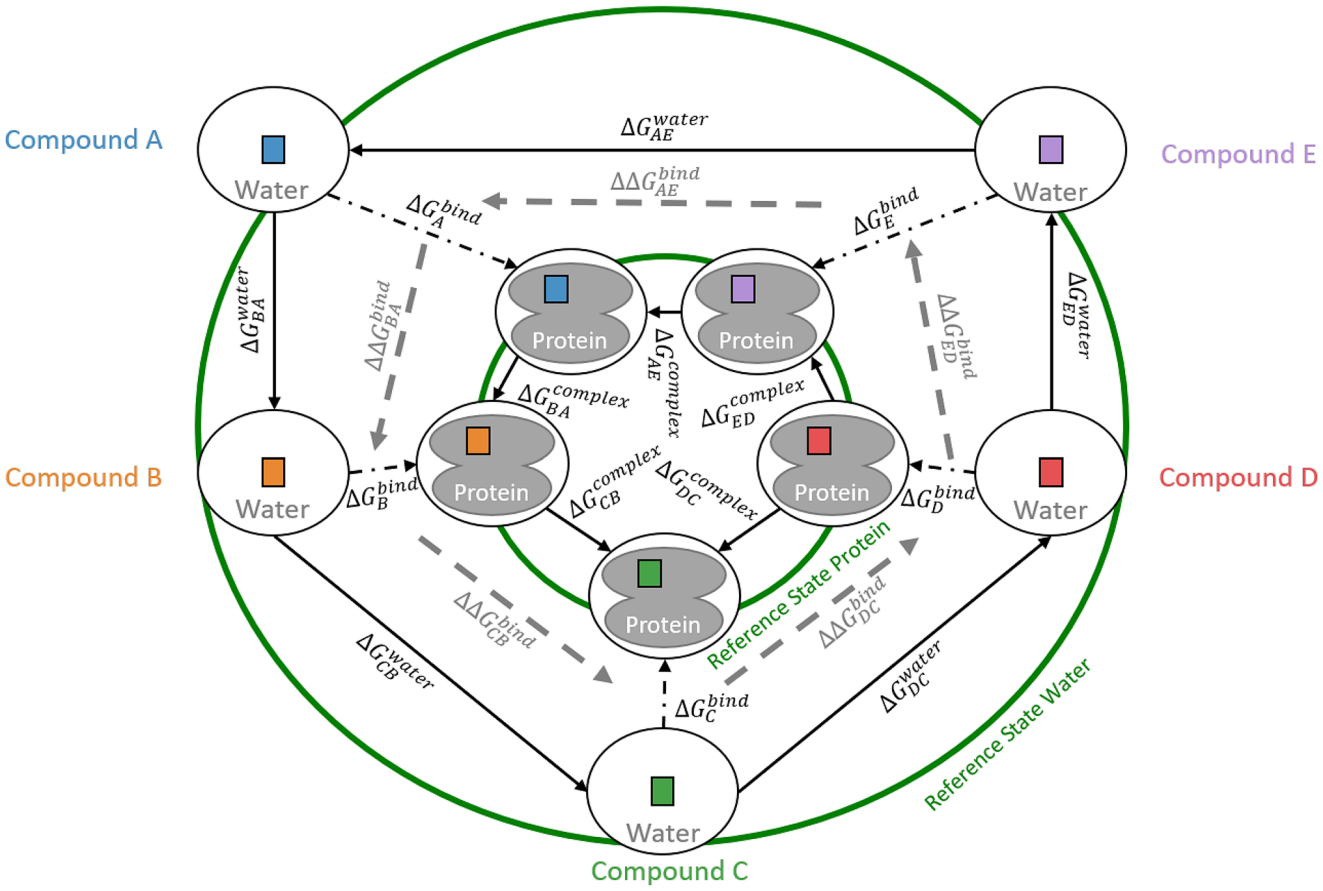
State graph to calculate relative binding free energies, where the nodes represent specific compounds *A*–*E* in a particular environment (water/protein). The connecting (directed) edges describe the transformations from one end state to another. The dashed-dotted arrows denote the (absolute) binding free energy of compound *i* to the protein, $\varDelta G_{i}^\text{bind}$, whereas solid arrows indicate alchemical transformations between compound *i* to compound *j* in a given environment. From the resulting $\varDelta G_{ji}^\text{env}$, $\varDelta \varDelta G^\text{bind}_{ji}$ can be calculated (gray dashed arrows) and compared with the value obtained from the difference of the experimentally determined $\varDelta G_{i}^\text{bind}$. In pathway-dependent methods, each edge between two end states is calculated separately. With (RE-)EDS, all end states in a given environment can be considered simultaneously in a single simulation of a reference state (green circles)

Computational free energy calculations typically make use of thermodynamic cycles. For instance, to estimate the binding free energy of five compounds, a “state graph” can be constructed (Fig. 1), where the nodes represent the end states and the edges the free-energy differences between them. Although not impossible [5], the direct calculation of (absolute) binding free-energies ($\varDelta G^\text{bind}_i$) is generally very challenging to achieve computationally [1]. A simpler alternative is to calculate the alchemical free-energy differences between two compounds *i* and *j* in a given environment ($\varDelta G_{ji}^{env}$) and then compare the relative binding free energy $\varDelta \varDelta G^\text{bind}_{ji}$ with the difference of the $\varDelta G^\text{bind}_i$ obtained from experiment [6, 7],

1 $$\begin{aligned} \varDelta \varDelta G^\text{bind}_{ji} = \varDelta G_{ji}^{\text{protein}} - \varDelta G_{ji}^{\text{water}} = \varDelta G^\text{bind}_j - \varDelta G^\text{bind}_i \end{aligned}$$

Conventional free-energy methods such as thermodynamic integration (TI) [8] and free-energy perturbation (FEP) [9] introduce a coupling parameter $\lambda$ to define a pathway from end state *i* ($\lambda =0$) to end state *j* ($\lambda =1$). In practice, simulations at discrete intermediate $\lambda$-points are performed to obtain converged free-energy differences.

If a (large) series of *N* compounds is investigated, the free-energy difference for all $(N(N-1))/2$ pairs of ligands would in principle have to be calculated. To reduce the computational cost, automatic schemes have been developed to identify the edges in the state graph (Fig. 1) with the smallest perturbations such that all nodes (for a given environment) are connected [10–12]. It is thereby important to include some cycles as cycle closure is a frequently used measure to assess convergence. Nevertheless, manual optimizations may sometimes be required to determine the best sampling strategy [13]. Furthermore, calculating only a subset of the edges leads to a larger uncertainty in the estimated free-energy difference for pairs that are no longer directly connected. As $\varDelta \varDelta G^\text{bind}_{ji}$ values are often relatively small, the increased uncertainty may negatively impact the usefulness of such calculations in practical applications.

An attractive and more efficient alternative to path-dependent methods is to simulate a reference state, which includes all *N* end states simultaneously, without the specification of pathways (green rings in Fig. 1). Such a reference state is provided by the enveloping distribution sampling (EDS) [14–17] method. The EDS reference state can be tuned for optimal sampling with parameters. Note that cycle closure is guaranteed by definition in this approach. In order to enhance sampling further, combinations of EDS with enhanced sampling methods were developed such as replica-exchange EDS (RE-EDS) [18–20] and accelerated EDS [21, 22].

In this study, we present an improved automated workflow for RE-EDS simulations that was restructured into two phases. The first phase aims to automatically estimate method parameters that otherwise had to be provided by the user. The second phase automatically optimizes the estimates from the first phase to retrieve a robust parameter set. The final production phase calculates the relative binding free energies of multiple ligands from a single simulation per environment. The robustness and versatility of the RE-EDS workflow are demonstrated on a series of five inhibitors of human checkpoint kinase 1 (CHK1) [23]. These ligands were selected by Wang et al. [24] as a challenging benchmarking set for FEP calculations since the changes between these ligands exemplify different types of core-hopping transformations (i.e. ring size change, ring opening/closing, and ring extension). Special soft bond-stretching terms were developed to be able to handle these transformations [24]. In contrast to other methods, no such special soft bonds are required with RE-EDS as we can use a “dual topology” approach [17] in a straightforward manner.

## Theory

### Enveloping distribution sampling (EDS)

In EDS, free-energy differences between multiple end states are obtained by sampling a reference-state Hamiltonian, i.e. without the definition of specific alchemical paths [14, 15, 17]. Given *N* end states, the potential energy function *V* of the EDS reference state *R* is defined as,

2 $$\begin{aligned} V_R(\mathbf{r}; s, \mathbf{E}^R)= - \frac{1}{\beta s}\ln \left[ \sum ^N_{i=1}{e^{-\beta s \left( V_i(\mathbf{r})- E_i^R\right) }} \right] , \end{aligned}$$

where $\beta = (k_\mathrm {B} T)^{-1}$ with $k_\mathrm {B}$ being the Boltzmann constant and *T* the absolute temperature. The smoothing parameter *s* and the energy offsets $\mathbf{E}^R$ were introduced to enable tuning of the reference state for optimal sampling of all end states [14, 15].

**Fig. 2:**
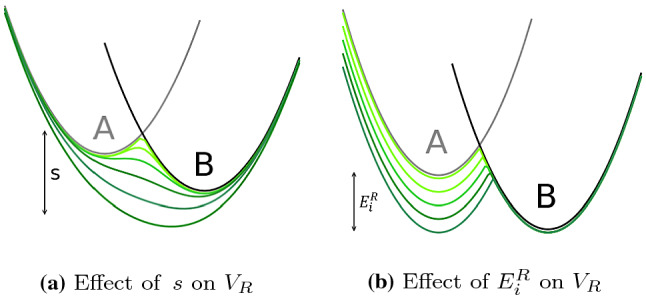
Schematic illustration of the effect of the two types of EDS reference-state parameters. **a** The smoothing parameter *s* decreases the barriers between the end states. If *s* is too small, an “undersampling” situation occurs with a global unphysical minimum. **b** The energy offsets $\mathbf{E}^R$ provide equal weighting to all end states in the EDS reference state. The figure was generated with Ensembler [27]

A smoothness parameter $s=1.0$ gives a reference potential-energy landscape that contains all the relevant minima of the end states. However, these might be separated by high barriers. For $s < 1$, the energy barriers between different end states $V_i$ are smoothed in the reference state $V_R$, increasing the transition rates between the different minima (Fig. 2a) [15]. However, if *s* is chosen too small, $V_R$ consists of a global unphysical minimum, which does not correspond to any of the end states. In the limit of $s\rightarrow 0$, all end states contribute equally to the potential-energy function of the reference state [25], which can lead to unphysical configurations. The situation with a too small *s* has been termed “undersampling” [17].

The energy offsets $\mathbf{E}^R$ are used to ensure equal weighting of all end states $V_i$ in $V_R$ (Fig. 2b). Note that the optimal values of *s* and $\mathbf{E}^R$ are not independent of each other (as can be seen in Eq. (2)) [15]. Different schemes have been proposed to determine optimal reference-state parameters [16, 17, 26], however, these are only applicable to systems with two end states.

The force on a particle *k* in the EDS reference state is calculated as [15],

3 $$\begin{aligned} \mathbf{f}_k(t)=-\frac{\partial V_R(\mathbf{r}; s, \mathbf{E}^R)}{\partial \mathbf{r}_k} = \sum ^N_{i=1}\frac{e^{-\beta s(V_i(\mathbf{r}) -E_i^R)}}{\sum ^N_{j=1}{e^{-\beta s (V_j(\mathbf{r})-E_j^R)}}} \left( -\frac{\partial V_i(\mathbf{r})}{\partial \mathbf{r}_k} \right) \,. \end{aligned}$$

For *s*-values close to one, the reference-state forces are dominated by the one end state, for which the current coordinates are most favourable, while the other end states give high energies and therefore contribute little (i.e. “dummy states”). For small *s*-values (undersampling situation), all end states contribute effectively to the forces, resulting in the global unphysical minimum.

The free-energy difference between two end states *A* and *B* can be calculated by employing the Zwanzig equation twice forming a path via the reference state *R* [9, 14, 15],

4 $$\begin{aligned} \varDelta G_{\text{BA}}&= \varDelta G_{\text{BR}} + \varDelta G_{\text{RA}} \nonumber \\&=-\frac{1}{\beta }\left( \ln \langle e^{-\beta (V_B-V_R)}\rangle _R - \ln \langle e^{-\beta (V_A-V_R )}\rangle _R\right) \end{aligned}$$

5 $$\begin{aligned}&= -\frac{1}{\beta } \ln \frac{\langle e^{-\beta (V_B-V_R)}\rangle _R}{\langle e^{-\beta (V_A-V_R)}\rangle _R}. \end{aligned}$$

### Replica-exchange EDS (RE-EDS)

**Fig. 3:**
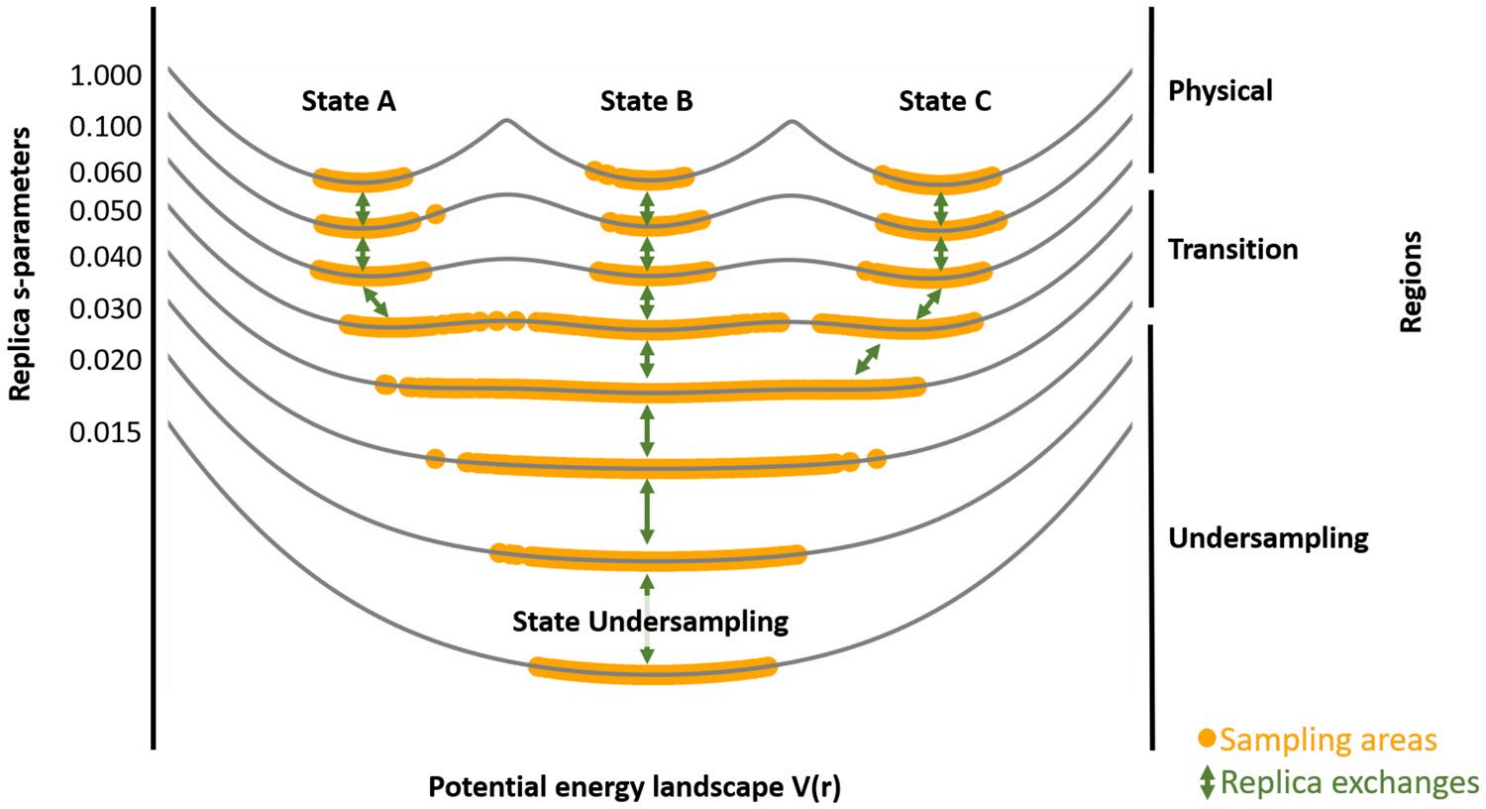
Schematic illustration of RE-EDS with three harmonic oscillators as end states (*A*, *B*, and *C*). Each replica differs by the *s*-parameter, generating reference states with a different degree of smoothness. Sampling of each replica is denoted with orange dots. Exchanges between the replicas are indicated with green arrows. The replica graph shows three regions: a “physical” region where *s* is close to 1, a transition region, and the “undersampling” region when *s* approaches zero. The figure was generated with Ensembler [27]

The recently introduced RE-EDS method [19, 20] is a type of Hamiltonian replica exchange [28, 29] with the smoothness parameter *s* as the exchange dimension ($1\geq s>0$), which was inspired from constant pH simulations by Lee et al. [18, 30]. The approach is shown schematically in Fig. 3. RE-EDS does not require a single (optimal) *s*-value. Instead, enhanced sampling is achieved by exchanging between the replicas with different smoothness levels. This simplifies the parameter choice problem and thus, the method can be applied to systems with more than two end states [19, 20].

For the pairwise exchanges between neighboring replicas *k* and *l*, a Metropolis-Hastings criterion [31] is used [19, 29],

6 $$\begin{aligned} p_{k,l}= & {} min\left( 1, \exp \left[ -\beta ((H_{R}(\mathbf{r}_k; s_l)+H_{R}(\mathbf{r}_l; s_k))\right. \right. \nonumber \\&\left. \left. -(H_{R}(\mathbf{r}_l; s_l)+H_{R}(\mathbf{r}_k; s_k)) \right] \right) , \end{aligned}$$

where $H_{R_k}$ and $H_{R_l}$ are the reference-state Hamiltonians of the respective replicas, $\mathbf{r}_k$ and $\mathbf{r}_l$ are the current coordinates of the replicas.

Replicas are placed between $s=1.0$ and a lower bound of *s*, where the reference state is in undersampling. The replicas with low *s*-values facilitate the transitions between the low-energy regions of the different end states. Especially for systems with slowly adapting environments (e.g. protein binding pockets), regions in *s*-space with very low acceptance probability can occur. Thus, to ensure sufficient exchanges between all pairs of replicas, a local variant of the round-trip time optimization algorithm [32, 33] was developed to optimally place the replicas in *s*-space [20]. It was found that a single set of energy offsets can be used for all replicas [19]. However, it is important that these energy offsets are chosen well to avoid “leakage” effects, resulting in one or more end states not being properly sampled [19]. The final free-energy differences are estimated from the replica at $s=1.0$, which represents the physical minima of the end states.

### Automatic parameter optimization

**Fig. 4:**
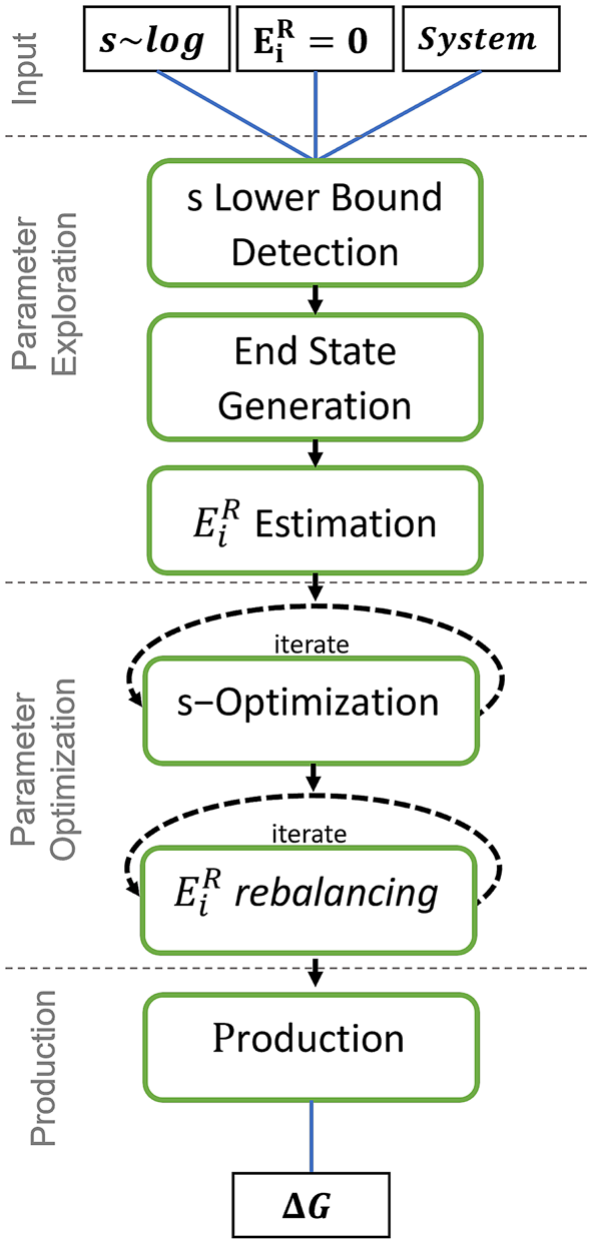
The RE-EDS workflow can be split into four steps: (1) Input stage with energy offsets set to $E_i^R=0$ and a set of *s*-parameters logarithmically distributed between 1 and $10^{-5}$; (2) Parameter exploration to determine the lower bound for *s*, to obtain equilibrated coordinates for each end state, and to estimate initial energy offsets with the PEOE scheme [19]; (3) Parameter optimization to improve the *s*-distribution with the N-LRTO algorithm [20] and the state sampling with energy offset rebalancing; (4) Production run and calculation of the free-energy differences

To facilitate the determination of the energy offsets and *s*-parameter distribution, we have extended and further automated the previous [20] RE-EDS workflow (Fig. 4).

The initial input for a system with *N* end states consists of a prepared EDS system (i.e. topology, perturbation topology, initial coordinates, and distance restraints), a list of energy offsets of length *N* with $E_i^R = 0; ~ \forall ~ i \in [1,...,N]$, and a list of *s*-parameters, which are logarithmically distributed in the range $s_i \in [1, 10^{-5}]$. Typically, we use 21 initial *s*-values.

The parameter exploration consists of three substeps: (i) determining the lower bound for the *s*-distribution (newly introduced), (ii) obtaining optimized coordinates within the EDS set-up for each end state (newly introduced), and (iii) estimation of an initial set of energy offsets (as done previously in Ref. [19]).

To enable sampling of all end states at $s=1.0$, some replicas have to be in undersampling to facilitate transitions. However, for efficiency reasons (and numerical stability) the number of replicas *M* in undersampling should be small and the lowest *s*-value should be as high as possible. From a short simulation with the initial *s*-distribution between $[1, 10^{-5}]$, the highest smoothing parameter $s_{M_\mathrm {us}}$ at which undersampling still occurs is determined and used in the following as a lower bound for the *s*-distribution. The *s*-distribution for the next step is then defined by logarithmically distributed replicas between $s=1.0$ and the automatically determined lower bound.

Optimized coordinates for each end state in the EDS setup can be obtained from short parallel simulations, where one end state in turn is favoured by setting an arbitrarily large energy offset for this state. The optimized coordinates allow the user to start RE-EDS simulations from different end states and are needed for the subsequent parameter optimization.

In the last substep, the $E_{\text{i}}^{\text{R}}$ estimation, the previously developed parallel energy offset estimation (PEOE) [19] scheme is used to estimate the initial set of energy offsets. This is done based on a short simulation with the initial parameters. For each replica *k* in the undersampling region, the energy offsets are extracted using [19],

7 $$\begin{aligned} E_{i}^{R}(new)=-\frac{1}{\beta }\ln \Big < e^{-\beta \big (V_i(\mathbf{r})-V_R(\mathbf{r}; s_{k},\mathbf{E}^{R}(old))\big )}\Big >_{R(s_{k},\mathbf{E}^{R}(old))} . \end{aligned}$$

The energy offsets that were extracted in parallel for the *k* replicas are subsequently averaged and used as initial set of energy offsets. These energy offsets should provide a first solution that is close to the optimal choice of energy offsets, which leads to an optimal state sampling of all end states in the RE-EDS simulation. As the initial energy offsets are obtained from the replicas in undersampling, they may not be exactly optimal and require fine-tuning in the next phase.

In the second step of the RE-EDS workflow, first the *s*-distribution is optimized and subsequently the energy offsets are fine tuned. The *s*-distribution is improved by minimizing the round-trip time $\tau$ and increasing the number of round-trips with the multistate local round-trip time optimization (N-LRTO) algorithm [20]. The optimization is performed in an iterative manner with short simulations. This step is required as exchange bottlenecks between two replicas might occur leading to a very slow round trip time or to no round trips at all. In the N-LRTO algorithm, new replicas are inserted in each iteration by linear interpolation in the *s*-regions with exchange bottlenecks, while the replica positions of the previous iteration are retained. Adding replicas theoretically increases the round-trip time due to a longer path between the top and bottom replicas. However, the addition of intermediate replicas also increases the exchange probability between neighboring replicas, thus reducing the round-trip time. With the optimization algorithm, we aim to determine the balance between the length of the replica path and the likelihood of exchange between replicas for minimal round-trip time. The exchange bottlenecks are identified for each end state separately (i.e. multistate). The number of replicas added can be chosen by the user. The iteration is stopped when the average round-trip time ${\overline{\tau }}$ converges. The N-LRTO variant is needed for systems for which severe bottlenecks are observed with the initial logarithmic *s*-distribution (e.g. protein binding pockets). For systems with smaller perturbations, the global multistate variant (N-GRTO) [20] can be more efficient as this algorithm re-distributes the replicas in *s*-space according to the exchange statistics. In this study, we started with the same number of replicas as used for the PEOE scheme above and added four replica positions per iteration in the N-LRTO algorithm.

After optimizing the number of round trips and $\tau$, the distribution of the state sampling is improved. To reach the ideal situation that each end state is sampled to an equal amount, the initial energy offsets need to be fine tuned, while keeping the round trips approximately constant. For this, we introduce here the energy offset rebalancing scheme. To avoid overshooting, a correction factor is calculated and applied iteratively,

8 $$\begin{aligned} \varDelta E^{corr}_i = - \frac{1}{\beta } \ln \left( \frac{f_i^{\text{mc}}+c}{f^{\text{mc,ideal}}_{i}+c} \right) , \end{aligned}$$

where $f_i^{\text{mc}}$ is the current sampling fraction (or estimated probability) of an end state contributing to $V_R$, and $f^{\text{mc,ideal}}$ is the ideal sampling fraction (see “Analysis” section). To make the approach more robust, a pseudo count *c* is introduced to avoid singularities with zero sampling, which is defined as,

9 $$\begin{aligned} c = \frac{f^{\text{mc,ideal}}}{x}, \end{aligned}$$

with the intensity factor *x*. The default of the pseudo count was chosen to result in a maximal correction of $\varDelta E^{corr}_i=8.43$ kJ mol^−1^, corresponding to a minimum 30-fold reduced sampling compared to the expected optimal sampling.

After optimizing the RE-EDS parameters, the production run is performed for a chosen length. The free-energy differences are subsequently calculated using the replica at $s=1.0$ with Eq. (5).

#### Starting state mixing

The sampling in RE-EDS simulations can be further improved by using starting coordinates for the replicas corresponding to the different end states (i.e. replica 1 starts in a low-energy configuration for end state 1, replica 2 in a low-energy configuration for end state 2, etc.). This technical approach is called “starting state mixing” (SSM) in the following and is also used with Hamiltonian replica-exchange TI calculations (see e.g. [34, 35]). The optimized coordinates obtained in the parameter exploration step can be used for SSM. We compare RE-EDS simulations with SSM and with a single set of starting coordinates (abbreviated as 1SS).

#### Analysis

Three types of metrics were used to quantify the sampling in RE-EDS simulations. The first metric determines for each end state *i* the sampling fraction where it is maximally contributing to the reference state, i.e. $f_i^{\text{mc}}$. A maximally contributing state is defined as the end state with the lowest potential energy minus its energy offset in a frame. As can be seen in Eq. (3), maximally contributing end states have the largest impact on the reference-state sampling at a given time point.

Optimal sampling in a RE-EDS system is achieved when all end states are sampled as maximally contributing states to an equal extent at $s=1.0$, i.e.

10 $$\begin{aligned} f_{i}^{\text{mc,ideal}} = \frac{1}{N} ~,\quad \forall ~ i~\in ~ \{1, ..., N\} \end{aligned}$$

The second metric is the estimated sampling fraction of “physical occurrence” of an end state *i*, i.e. $f_i^{\text{occur}}$. As a result of phase-space overlap with the current maximal contributing end state, other end states in the EDS system might be sampled simultaneously. An end state is counted as “occurred” when its potential energy is below the threshold $V_i \le T_{i}^{\text{phys}}$ at a time point *t*. These thresholds are estimated during the second substep of the parameter exploration phase. If end states show no phase-space overlap, $f_i^{\text{occur}}$ will be (nearly) the same as $f_i^{\text{mc}}$.

Undersampling is detected with a third metric using the thresholds $T_{i}^{\text{us}}$. These thresholds are determined in the first substep of the parameter exploration phase from the simulation with the lowest *s*-value. If all end states have a potential energy below their respective $V_i - E^R_i \le T_{i}^{\text{us}}$, the current frame is characterized as undersampling [19].

## Methods

### Model system

**Fig. 5:**
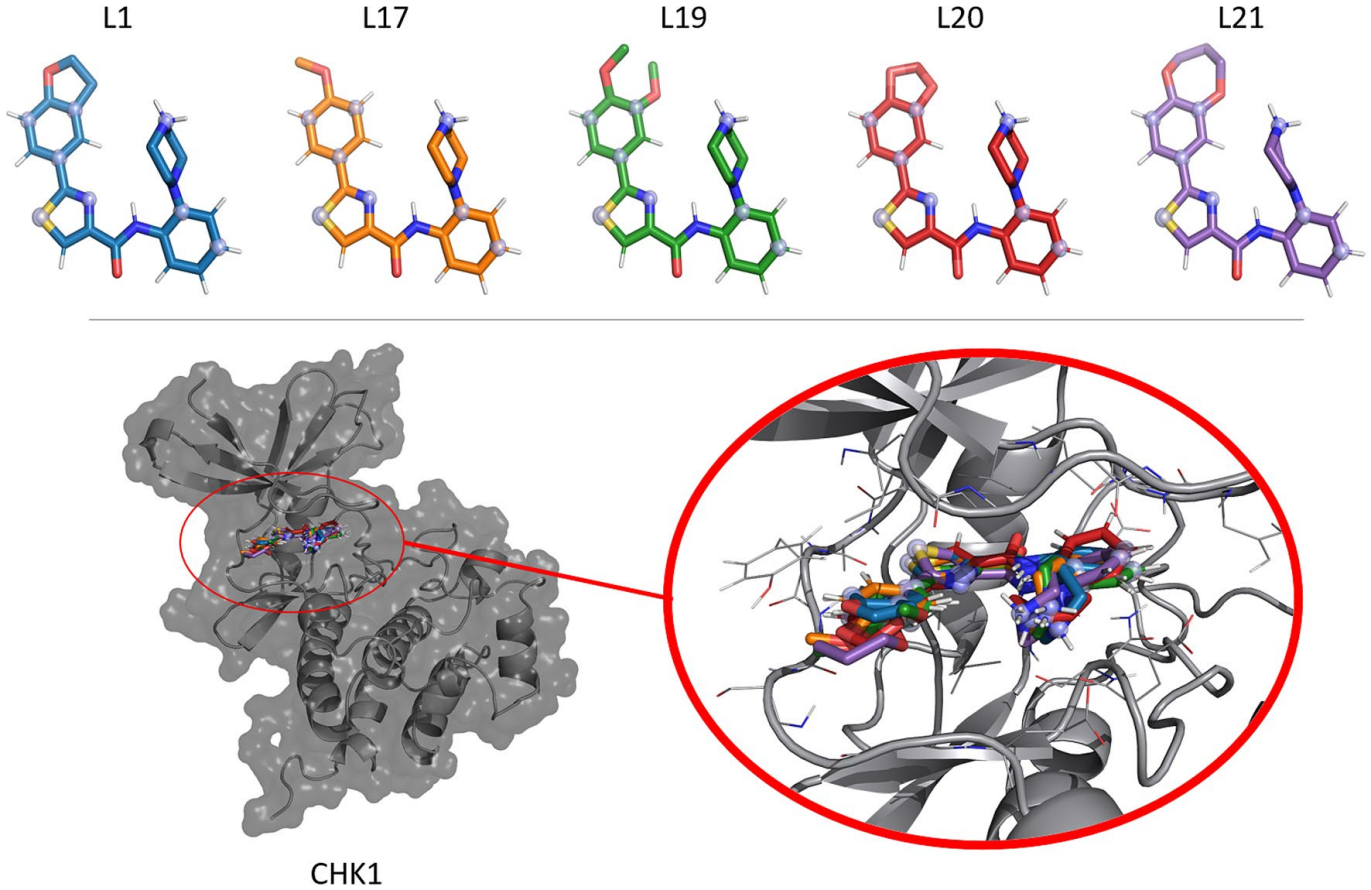
(Top): 3D depiction of the five CHK1 inhibitors L1, L17, L19, L20, and L21 (numbering according to Ref. [23]). The selected locations of the distance restraints are indicated by the silver spheres. (Bottom): CHK1 protein in complex with the ligand bundle (PDB ID:3U9N)

To showcase the performance of RE-EDS, a system of five inhibitors (L1, L17, L19, L20 and L21) of checkpoint kinase 1 (CHK1) taken from Ref. [23] was chosen (Fig. 5). The numbering of the compounds is according to Ref. [23]. The same system was studied in Ref. [24] as part of a series of scaffold hopping systems. Although the five ligands share a common substructure, they were considered to exemplify different types of core-hopping transformations (i.e. ring size change, ring opening/closing, ring extension) and R-group modifications [24].

For the protein, the GROMOS 54A7 force field [36] was used. For the ligands, topologies were generated using the parametrization by the ATB server [37] as an initial guess. The bonded terms were manually harmonized and adjusted to match the parameterization of similar functional groups in the GROMOS 54A7 force field. Partial charges were generated with our previous machine learning approach [38] ($\epsilon$ = 4) and manually arranged into charge groups. The input files can be retrieved from: https://github.com/rinikerlab/reeds/tree/main/examples/systems.

### System preparation

The crystal structure of CHK1 in complex with ligand L1 (PDB ID:3U9N) was used as starting structure. The initial coordinates for ligands L17, L19, L20, L21 were generated with the ConstrainedEmbed() functionality in the RDKit [39], where the common substructure was kept fixed in the crystal conformation. The coordinates of each ligand and those of the protein were subsequently energy minimized in vacuum using the steepest descent [40] approach implemented in the GROMOS software package [41].

A “dual topology” approach was used for the RE-EDS simulations, i.e. each ligand is present in the system separately [17]. Thus, each end state comprises of one active ligand and $N-1$ inactive (dummy) ligands. To avoid spatial drifting of the dummy ligands, eight distance restraints per ligand pair were defined within the common substructure (Fig. 5) to connect all ligands in a ring with the help of the RestraintMaker program (https://github.com/rinikerlab/restraintmaker) (order: -L1-L17-L19-L20-L21-). The reference distance was set to 0.0 nm and the force constant to 1000 kJ mol$^{-1}$ nm$^{-2}$. The combined topology file was generated with the program prep_eds in the GROMOS++ [42] package. The EDS system was solvated in a cubic box of simple-point-charge (SPC) [43] water (resulting in 1’848 solvent molecules for the ligands in water and 15,639 solvent molecules for the protein-ligands complex). An energy minimization was carried out with the steepest descent algorithm [40], where all solute atoms were position restrained with a force constant of 25,000 kJ mol$^{-1}$ nm$^{-2}$.

### Simulation details

All simulations were performed with the GROMOS software package [41] (freely available on http://www.gromos.net). The equilibrations and production runs were carried out under isothermal-isobaric (NPT) conditions using the leap-frog integration algorithm [44] and a time step of 2 fs. Bond lengths were constrained with SHAKE [45] using a tolerance of $10^{-4}$. The nonbonded contributions were calculated with a twin-range scheme using a short-range cutoff of 0.8 nm and a long-range cutoff of 1.4 nm. The electrostatic nonbonded contributions beyond the long-range cutoff were calculated with the reaction-field [46] approach and a dielectric permittivity of 66.7 [47] for water.

The temperature was kept constant at 300 K using the weak coupling scheme [48] and a coupling time of 0.1 ps$^{-1}$. The pressure was kept at 1.031 bar (1 atm) with the same type of algorithm and a coupling time of 0.5 ps and an isothermal compressibility of $4.575 \times 10^{-4}$ (kJ mol$^{-1}$ nm$^{-3}$)$^{-1}$. Rotation and translation of the center of mass of the simulation box were removed every 2 ps. Energies were written to file every 20 steps and coordinates every 5000 steps. In the RE-EDS simulations, replica exchanges was attempted every 20 steps.

### RE-EDS workflow

The new Python code to manage the RE-EDS workflow, including the analysis steps, can be retrieved from: https://github.com/rinikerlab/reeds. The workflow starts with the energy-minimized coordinates of the EDS system (all *N* ligands plus environment, maximally contributing end state is L20) into the parameter exploration step, which is used as equilibration phase. A RE-EDS simulation of 0.2 ns length was performed with 21 logarithmically distributed replicas between $s=1.0$ and $10^{-5}$ and all energy offsets set to zero. The thresholds $T_{i}^{\text{us}}$ were estimated from replicas with very low *s*-values. Undersampling was observed when each end state occurred with a fraction $f_{i}^{\text{occur,us}} \ge 0.75$ during the simulation period. To be conservative, the lower bound for the following steps was set to the *s*-value two levels below the highest replica with undersampling.

To optimize the coordinates of the system for each end state, an EDS simulation of 2 ns length was performed for each end state *i* with $s=1.0$ and $E^R_i=500$ kJ mol$^{-1}$ while the energy offsets of all other end states were set to $-500$ kJ mol$^{-1}$. L20 was the initial maximally contributing end state in the starting configuration. The coordinates were considered to be optimized when the desired end state was constantly sampled as the maximally contributing state in the last 30% of the simulation.

To determine the energy offsets, a 1.5 ns RE-EDS simulation was carried out with 12 logarithmically distributed replicas for the ligands in water and 17 for the protein-ligands complex between $s=1.0$ and the lower bound (determined above). The first 0.4 ns of the simulation were discarded as equilibration. This simulation was performed in two manners: (i) using the final coordinates from the lower-bound determination as starting configuration for all replicas (1SS approach), or (ii) using the different optimized coordinates from the previous substep for the replicas in an alternating manner (SSM approach). For the PEOE [19] scheme, the following parameters were used: fraction $f_{i}^{\text{us}} \ge 0.9$ and the potential thresholds determined in the lower bound exploration $T_{i}^{\text{us}}$.

The iterative optimization of the *s*-distribution with the N-LRTO [20] algorithm was started with the energy offsets and the final coordinates of the previous substep. Four replicas were added per iteration. The simulation length of the first iteration was 0.5 ns, and subsequently increased by 0.5 ns at each iteration until a maximum length of 1.5 ns was reached.

The iterative optimization of the $f_i^{\text{mc}}$ distribution was carried out with the described scheme. The scheme used short 0.5 ns simulations, and adjusted in each step the energy offsets $E^R$ with a pseudo-count intensity factor $x = 30$.

The optimization was considered converged here when all end states were sampled as maximally contributing states at $s=1.0$, the number of round trips per ns was above zero, and the improvement of the round-trip time was below ${\overline{\tau }}/nRT < 0.5$ ns.

The production run with constant reference-state parameters was performed for 3.5 ns.

### Simulation of single states

The input coordinates for the simulations of the individual end states were extracted from the RE-EDS starting coordinates and subsequently energy minimized. Next, a production run of 4 ns was performed.

### Analysis

Free-energy differences were calculated with the program dfmult from the *GROMOS++* [42] package. Statistical analysis and handling of the workflow steps are based on the Python packages pandas [49], Matplotlib [50], NumPy [51], SciPy [52], and PyGromosTools [53].

## Results and discussion

The chosen model system of five inhibitors of CHK1 kinase exemplifies different core-hopping transformations (i.e. ring size change, ring opening/closing, ring extension) and R-group modifications [24], increasing the complexity compared to the systems previously studied with RE-EDS. Furthermore, the performance can be directly compared to the results obtained with FEP+ and OPLS3 in Ref. [24] as well as with QligFEP results in Ref. [13].

### Parameter exploration and parameter optimization

The RE-EDS workflow was started by estimating the lower bound for the *s*-distribution. Using the above mentioned undersampling criterion (see “Methods” section), a lower bound of $s=0.01$ was determined for the protein-ligands complex and $s=0.0056$ for the ligands in water.

Optimized coordinates were obtained for all five ligands, as verified by comparing the potential-energy distribution from the EDS simulation with the one extracted from a standard MD simulation of the respective ligand (Fig. S1 in Supporting Information). From these same steps, the potential-energy thresholds for the occurrence sampling ($T_{i}^{\text{phys}}$) and undersampling ($T_{i}^{\text{us}}$) were determined.

The energy offsets $\mathbf{E}^R$ were estimated from a short RE-EDS simulation with the PEOE [19] scheme and are listed in Table 1. For $s=1.0$, the energy offsets should ideally be equal to the free energy of the corresponding state (i.e. $\varDelta E^R_{ji} = \varDelta G_{ji}$) such that the partition function of the reference state is the sum of the partition functions of the end states [15]. Therefore, the comparison between the relative estimated energy offsets in water and in complex ($\varDelta \varDelta E^R_{ji} = \varDelta E^R_{ji,\text{complex}} - \varDelta E^R_{ji,\text{water}}$) and the relative binding free energy $\varDelta \varDelta G^\text{bind}_{ji}$ can be used to (roughly) assess the quality of the estimated energy offsets. As shown in Fig. S2 in Supporting Information, the energy offsets estimated from the SSM simulations are in better agreement with the experimental relative binding free energies than those estimated from the 1SS simulations.

**Table 1:** Energy offsets $\mathbf{E^R}$ estimated from a short RE-EDS simulation using the PEOE [19] scheme

Ligand	Water		Complex	
	RE-EDS 1SS [kJ mol$^{-1}$]	RE-EDS SSM [kJ mol$^{-1}$]	RE-EDS 1SS [kJ mol$^{-1}$]	RE-EDS SSM [kJ mol$^{-1}$]
L1	0.0	0.0	0.0	0.0
L17	$11.07 \pm 7.61$	$17.81 \pm 0.69$	$20.03 \pm 5.04$	$18.19 \pm 3.43$
L19	$-9.38 \pm 6.85$	$-12.37 \pm 5.23$	$-2.09 \pm 1.56$	$2.4 \pm 1.56$
L20	$-53.15 \pm 2.95$	$-56.01 \pm 13.67$	$-58.73 \pm 4.87$	$-52.2 \pm 2.6$
L21	$-76.75 \pm 5.79$	$-69.15 \pm 3.74$	$-77.29 \pm 3.12$	$-77.9 \pm 3.4$

The errors indicate the standard deviation over the different replicas in undersampling. All energy offsets were calculated relative to ligand L1. The starting coordinates were selected following the 1SS or the SSM approach (see “Theory” and “Methods” sections)

The optimization of the *s*-distribution was performed with the N-LRTO [20] algorithm, thereby minimizing the average round-trip time ${\overline{\tau }}$ in the replica graph. For the 1SS approach in the complex, four optimization iterations were used. For the other systems, three iterations were used.

**Fig. 6:**
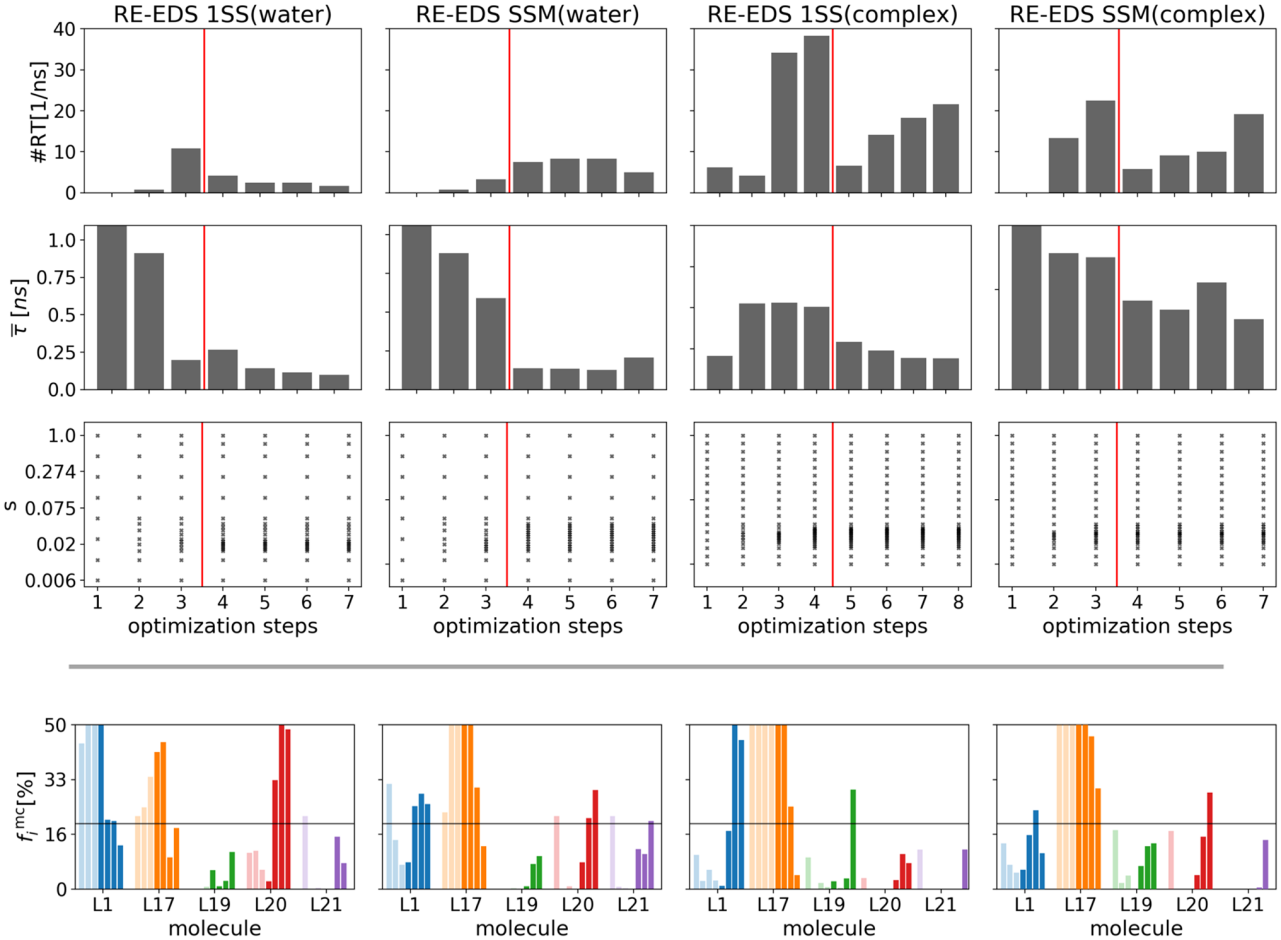
Optimization steps of the *s*-distribution with the N-LRTO [20] algorithm followed by the energy offset rebalancing scheme (start indicated by the red horizontal line). The measured quality criteria were the number of round trips (1. row), the average round-trip time ${\overline{\tau }}$ (2. row), the placement of the replicas in *s*-space (3. row), and the sampling fractions of maximally contributing states $f_{i}^{\text{mc}}$ (4. row). The light colored bars of $f_{i}^{\text{mc}}$ indicate *s*-optimization iterations, whereas the fully colored bars indicate energy offset rebalancing steps

In the first iteration, the total number of observed round trips was very low or zero for all approaches. In the following iterations, this quantity increased, and the average round-trip time decreased for all simulations (Fig. 6). The number of round trips was generally smaller in the complex than in water due to a more pronounced gap region [20]. Already after the second iteration, the round-trip time was reduced in all approaches. The improvement of the ${\overline{\tau }}$ over the iterations can also be seen in Fig. S3 in Supporting Information. As can be seen in the third row of Fig. 6, the optimization algorithm increases the density of the replicas around $s = 0.041$, where the major gap region lies.

The *s*-optimization was stopped after a sufficiently high number of round trips and low round-trip time was reached. This resulted in 20 replicas for the ligands in water after three *s*-optimization iterations. For the protein-ligands complex, the fourth *s*-optimization iteration was chosen for the 1SS approach, and the third iteration for the SSM approach, resulting in 29 and 25 replicas, respectively. The average round-trip time after convergence was ${\overline{\tau }} = 0.4 \pm 0.2$ ns for all simulations.

After the *s*-optimization, the energy offset rebalancing scheme was applied to improve the state sampling.

During the rebalancing steps, no further replicas were added to the *s*-distribution. It is essential for the success of the rebalancing scheme that round trips occur. Therefore, the number of round trips and average round-trip time were monitored. In all systems, the number of round trips and ${\overline{\tau }}$ remained relatively stable over the four rebalancing steps. For the RE-EDS 1SS approach in water, the number of round trips slightly decreased but never dropped to zero.

Across the optimization steps, also the sampling of the end states as maximally contributing states at $s=1.0$ was monitored. During the *s*-optimization, some end states “vanish” and are no longer sampled as maximal contributing states. This leakage effect can occur when the initially estimated $E^{\text{R}}$ are not exactly optimal [19]. With energy offset rebalancing, the sampling of each end state can be recovered, and the sampling distribution approaches the ideal case. After rebalancing, all end states showed a $f_i^{\text{mc}} > 0$ and the mean absolute deviation of the sampling distribution from ideal decreased from 20–25% to approximately 7–12% (Fig. S4 in Supporting Information).

### Free-energy calculation

After successfully optimizing the RE-EDS parameters, the production runs were performed for 3.5 ns.

**Fig. 7:**
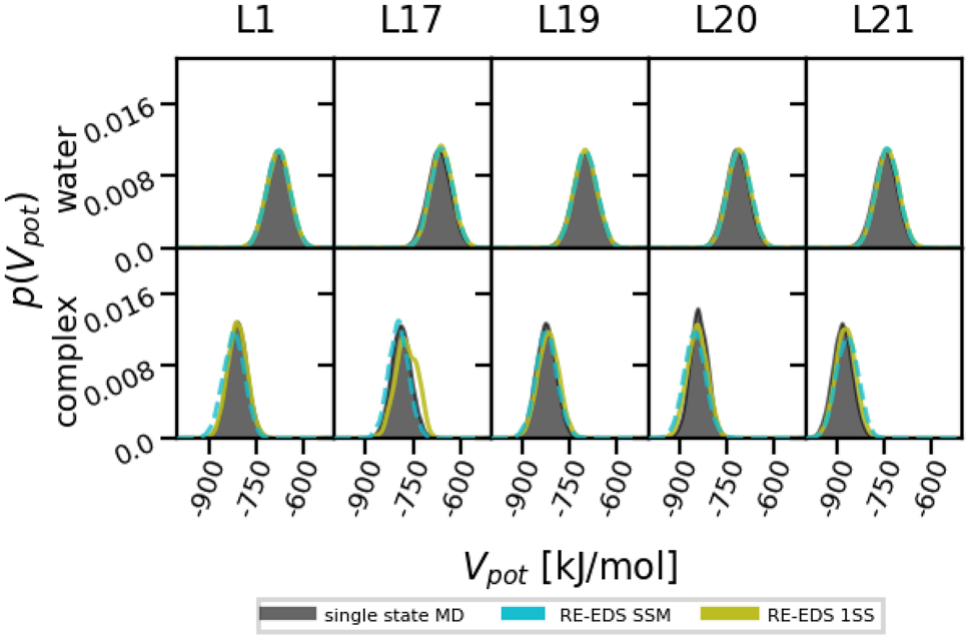
Comparison of the Boltzmann reweighted potential-energy distributions obtained from standard MD simulations of a given end state (black) and from the RE-EDS production runs with the 1SS (green) and SSM (turquoise, dashed) approaches

Both in water and in complex, the potential-energy distributions of the end states generally agree well with the corresponding distributions from the standard MD simulations of the single end states (Fig. 7). Only for the 1SS approach in the complex, a deviation can be seen for L17, with a slight shift to higher potential energies. This is due to insufficient sampling of L17 in this case (see below). The analysis of the maximally contributing end states at $s=1.0$ shows that in water all end states were sampled close to the ideal equal distribution (Fig. S5 in Supporting Information). In the simulation of the protein-ligands complex, there are still differences in sampling. Especially with the 1SS approach, L19 is generally sampled too often, while L17 is not sampled enough. The situation is improved with the SSM approach. Comparing $f_i^{\text{occur}}$ and $f_i^{\text{mc}}$ in Fig. S5 indicates that the end states in the CHK1 system are clearly separated (i.e. no phase-space overlap).

**Fig. 8:**
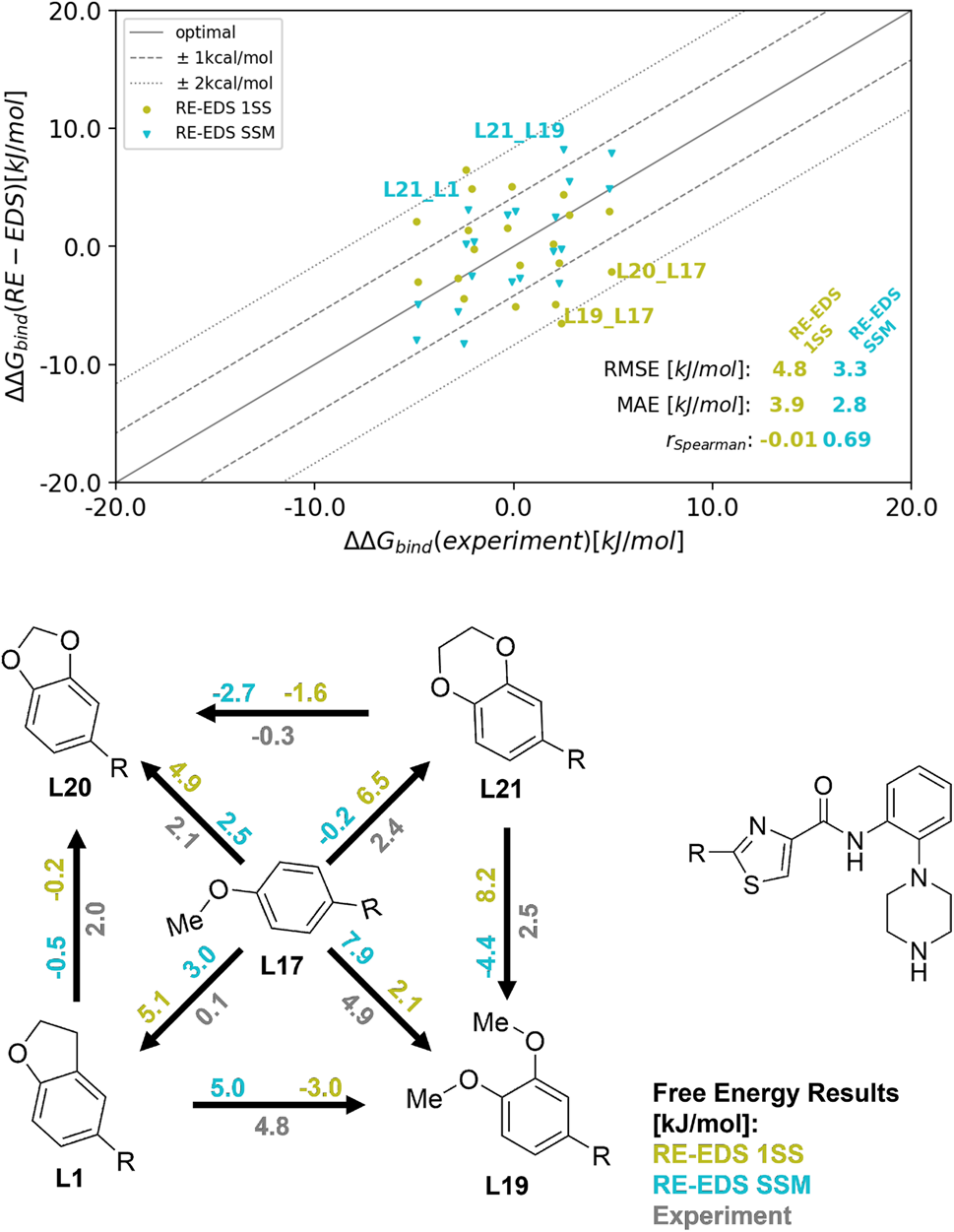
Free-energy differences estimated from the production run of 3.5 ns length. (Top): Comparison between the experimental and calculated $\varDelta \varDelta G^\text{bind}_{ji}$ using RE-EDS 1SS and RE-EDS SSM. The results were calculated with all possible pairwise transformations (forward and backward). (Bottom): Graphical representation of the $\varDelta \varDelta G^\text{bind}_{ji}$ results with structures, inspired by the one in Ref. [24]

From the replica at $s=1.0$, the free-energy differences were calculated using Eq. (5) and the resulting $\varDelta \varDelta G^\text{bind}_{ji}$ were compared with the experimental results taken from Ref. [23]. The results are shown graphically in Fig. 8 and numerically in Table 2. The individual free-energy differences are given in Table S3 in the Supporting Information. The RMSE with RE-EDS 1SS is 4.8 kJ mol$^{-1}$ and the MAE is $3.9\pm 2.8$ kJ mol$^{-1}$. The main deviations stem from ligand L17 in the RE-EDS 1SS approach, which can be explained by the insufficient sampling of this ligand in the complex (see Figs. 7 and S5 in Supporting Information).

The performance was substantially improved using the SSM approach with RE-EDS, giving an RMSE of 3.3 kJ mol$^{-1}$ and an MAE of $2.8 \pm 1.7$ kJ mol$^{-1}$. Only two values (L21–L11) and (L21–L19) deviate more than 4.184 kJ mol$^{-1}$ (i.e. 1 kcal mol$^{-1}$) from experiment. The Spearman correlation coefficient for RE-EDS 1SS is $r_{\text{Spearman}}=-0.01$ and for RE-EDS SSM $r_{\text{Spearman}}=0.69$.

Next, we assessed the convergence of the $\varDelta G_{ji}$ values as a function of simulation time (Fig. S6 in Supporting Information). For the RE-EDS 1SS approach, all free-energy differences appeared converged after 2.5 ns in water and after 2.7 ns in the complex. For the RE-EDS SSM approach, convergence was observed after 2.5 ns in water and after 2.9 ns in the complex.

By applying the RE-EDS methodology to the same system of five CHK1 inhibitors as studied by Wang et al. [24] and later on also Jespers et al. [13], a direct comparison with FEP+ and QligFEP is possible (Table 2). Note that the quality metrics were calculated over all possible pairs of ligands and in both directions, not only those directly calculated by FEP+ and QligFEP. For FEP+, we obtained an RMSE of 2.4 kJ mol$^{-1}$ and an MAE of $1.8 \pm 1.2$ kJ mol$^{-1}$ with a Spearman correlation coefficient of $r_{\text{Spearman}}=0.67$. Including cycle closure correction (CC) [24] reduced the RMSE to 2.1 kJ mol$^{-1}$ and the MAE to $1.9 \pm 1.0$ kJ mol$^{-1}$. The Spearman correlation coefficient increased to $r_{\text{Spearman}}=0.73$. Jespers et al. [13] reported free-energy differences with QligFEP as an average over ten independent replicas, each with significantly less simulation time per $\lambda$-window than in Ref. [24]. For QligFEP, an RMSE of 2.3 kJ mol$^{-1}$, an MAE of $2.0 \pm 1.2$ kJ mol$^{-1}$, and a Spearman coefficient of $r_{\text{Spearman}}=0.61$ was obtained.

Overall, the performance of RE-EDS SSM is comparable with the pairwise methods. The results with FEP+ CC and QligFEP showed a slightly higher accuracy compared to experiment, likely due to the different force fields used. The Spearman correlation coefficient is comparable with the other methods for the RE-EDS SSM approach.

**Table 2:** Relative binding free energies $\varDelta \varDelta G^\text{bind}_{ji}$ from experiment and calculated with the RE-EDS 1SS and RE-EDS SSM approaches

Ligands		Exp. [23]	FEP+ [24]	FEP+ CC [24]	QligFEP [13]	RE-EDS 1SS	RE-EDS SSM
*i*	*j*	[kJ mol$^{-1}$]	[kJ mol$^{-1}$]	[kJ mol$^{-1}$]	[kJ mol$^{-1}$]	[kJ mol$^{-1}$]	[kJ mol$^{-1}$]
L17	L1	0.1	− 3.6 ± 0.4	− 2.9 ± 1.0	− 1.6 ± 1.7	5.1 ± 0.8	3.0 ± 2.0
L19	L1	− 4.8	− 3.9 ± 0.3	− 4.0 ± 0.6	− 1.7 ± 2.0	3.0 ± 1.0	− 5.0 ± 0.1
L20	L1	− 2.0	− 2.5 ± 0.1	− 3.1 ± 1.0	− 1.3 ± 1.3	0.2 ± 0.9	0.5 ± 0.1
L21	L1	− 2.3	**− 3.4** ± **0.7**	**− 3.2** ± **1.3**	**− 0.1** ± **3.5**	− 1.4 ± 0.8	3.2 ± 0.1
L19	L17	− 4.9	− 1.4 ± 0.3	− 1.1 ± 1.0	**0.1** ± **2.6**	− 2.1 ± 0.6	− 7.9 ± 1.9
L20	L17	− 2.1	0.3 ± 0.4	− 0.1 ± 0.8	− 1.3 ± 2.3	− 4.9 ± 0.1	− 2.5 ± 1.9
L21	L17	− 2.4	− 1.1 ± 0.4	− 0.9 ± 0.9	**0.7** ± **2.6**	− 6.5 ± 0.1	0.2 ± 1.9
L20	L19	2.8	**0.8** ± **0.6**	**0.1** ± **1.3**	**− 0.4** ± **3.7**	− 2.7 ± 0.6	5.4 ± 0.1
L21	L19	2.5	− 0.1 ± 0.6	0.6 ± 0.1	0.6 ± 4.9	− 4.4 ± 0.6	8.2 ± 0.1
L21	L20	− 0.3	− 0.3 ± 0.8	− 0.6 ± 0.8	0.6 ± 1.1	− 1.6 ± 0.1	− 2.7 ± 0.1
RMSE			2.4 ± 0.3	2.1 ± 0.2	2.3 ± 0.38	4.8 ± 0.6	3.3 ± 0.3
MAE			1.8 ± 1.2	1.9 ± 1.0	2.0 ± 1.2	3.9 ± 2.8	2.8 ± 1.7
$r_{\text{Spearman}}$			0.67	0.73	0.61	− 0.01	0.69
$t_{simulation} [ns]$			640	640	51	171.5	157.5

For comparison, the results for FEP+ with and without cycle closure (CC) correction taken from Ref. [24] and the results for QligFEP taken from Ref. [13] are listed. The free-energy differences of directly simulated paths were used to infer not directly simulated free-energy differences (marked in bold). If multiple indirect paths were possible, their average was used. The errors for QligFEP were determined in Ref. [13] by calculating the standard deviation over ten replicas. For FEP+, the error of the results was taken from the used BAR [54] method and the FEP+ CC errors were obtained from the cycle closure analysis. For the RE-EDS approaches, the reported error is based on the statistical uncertainties of the $\varDelta G_{ji}^{env}$ values estimated using Gaussian error approximation [15]. The uncertainty estimate of the RMSE was obtained by a 100-fold bootstrapping approach

In terms of computational cost, the RE-EDS approach (with 3.5 ns per replica) resulted in about a quarter of the total simulation time (in ns) than reported for the FEP+ calculations in Ref. [24] (Table 2). Overall, the QligFEP approach is the one with the lowest simulation time consumption. A major advantage of the simultaneous simulation of multiple ligands in a single RE-EDS simulation is that all $N(N-1)/2$ transformations are sampled directly, leading to low statistical errors and removing the need for a state graph. This advantage increases with increasing number of ligands. The current workflow of RE-EDS uses a relatively large amount of simulation time for parameter optimization. Future work will focus on further optimizing the workflow to reduce the pre-processing time.

From the calculated relative binding free energies, $\varDelta G_{i}^{\text{bind}}$ can be obtained by using one experimental value as anchor point. This allows us to generate a ranking of the five ligands. To avoid any bias from the selected experimental anchor point, all possibilities were calculated and the resulting values averaged (Table 3). While the RMSE is generally low for all approaches (< 1 kcal mol$^{-1}$ = 4.184 kJ mol$^{-1}$), the ranking of the ligands as measured by $r_{\text{Spearman}}$ is not very good. This observation is not uncommon for ligand series with small differences in binding free energy [11, 55]. Note that the uncertainties of the individual values have increased compared to the relative binding free energies due to the anchoring and averaging procedure.

**Table 3:** Absolute binding free energies $\varDelta G_{i}^{\text{bind}}$ and ranking of the ligands derived from the relative binding free energies

Ligands	Exp. [23]	FEP+ [24]	FEP+ CC [24]	QligFEP [13]	RE-EDS 1SS	RE-EDS SSM
Molecule	[kJ mol$^{-1}$]	[kJ mol$^{-1}$]	[kJ mol$^{-1}$]	[kJ mol$^{-1}$]	[kJ mol$^{-1}$]	[kJ mol$^{-1}$]
L1	− 40.7	− 41.7 ± 1.7	− 41.7 ± 0.9	− 38.5 ± 1.5	− 40.0 ± 3.4	− 38.0 ± 2.0
L17	− 40.8	− 38.0 ± 1.0	− 38.2 ± 1.1	− 38.6 ± 1.3	− 33.7 ± 1.3	− 41.7 ± 2.3
L19	− 35.9	− 38.1 ± 0.9	− 38.3 ± 1.8	− 38.3 ± 1.0	− 37.6 ± 3.3	− 33.0 ± 2.0
L20	− 38.6	− 38.6 ± 1.6	− 38.3 ± 1.4	− 39.2 ± 1.7	− 40.4 ± 3.3	− 39.1 ± 2.3
L21	− 38.4	− 37.7 ± 1.4	− 37.8 ± 1.3	− 39.4 ± 1.9	− 42.4 ± 2.9	− 42.5 ± 1.4
RMSE		1.7 ± 0.4	1.7 ± 0.4	1.7 ± 0.4	3.8 ± 1.3	2.6 ± 0.6
MAE		1.3 ± 1.0	1.4 ± 0.9	1.4 ± 0.9	3.0 ± 2.3	2.2 ± 1.6
$r_{\text{Spearman}}$		0.20	0.10	− 0.21	− 0.40	0.30

The values were calculated from the relative binding free energies using an experimental binding free energy as anchor point, and then averaged over the five possibilities. The errors are standard deviations over the possible outcomes. For comparison, the results for FEP+ with and without cycle closure (CC) correction taken from Ref. [24] and the results for QligFEP taken from Ref. [13] are shown (calculated with the same procedure). The uncertainty estimate of the RMSE was obtained by a 100-fold bootstrapping approach

## Conclusion

This study reports the recent developments for the multistate free-energy method RE-EDS, which omits the definition of alchemical transition paths. The automated workflow for RE-EDS was improved in robustness, and was applied to estimate the relative binding free energies of five CHK1 inhibitors containing typical core-hopping transformations. This system was investigated previously with FEP+ and QligFEP, allowing for a direct comparison of RE-EDS with state-of-the-art pairwise free-energy methods. Using different starting configurations representing all end states (SSM approach) in the parameter optimization of the RE-EDS workflow improved the sampling, convergence, and the accuracy of the resulting free-energy differences. The performance of RE-EDS SSM was found to be comparable with FEP+ and QligFEP, and shows that RE-EDS with a “dual topology” approach can be readily applied to challenging ligand transformations like ring size change, ring opening/closing, and ring extension.

In terms of computational efficiency, the total production run time with RE-EDS (3.5 ns per replica) was about a quarter of that reported for FEP+ with this system. As multiple ligands are simulated simultaneously in a single RE-EDS simulation, this sampling enhancement will increase with increasing number of ligands. However, the pre-processing phase in the RE-EDS workflow currently uses a relatively large amount of simulation time. Making these steps more efficient will be addressed in future work. In addition, further automatization of the dual topology approach with distance restraints is ongoing.

## Supplementary Information

Below is the link to the electronic supplementary material.

Supplementary file1 (PDF 1276 KB)

## Data Availability

The Python code for the RE-EDS workflow is provided on Github https://github.com/rinikerlab/reeds and can be used with the current version of GROMOS, freely available from http://www.gromos.net. The input files for the simulations can be retrieved from https://github.com/rinikerlab/reeds/tree/main/examples/systems.
