# Supplementary material for: Relative free-energy calculations for scaffold hopping-type transformations with an automated RE-EDS sampling procedure
Source: J Comput Aided Mol Des. 2022 Jan 3;36(2):117–30. doi: 10.1007/s10822-021-00436-z (PMC8907147; doi:10.1007/s10822-021-00436-z)
Supplement: Supplementary file 1 — Supplementary file1 (PDF 1276 KB) [file 10822_2021_436_MOESM1_ESM.pdf]

SUPPORTING INFORMATION

Relative Free-Energy Calculations for Scaffold Hopping-Type Transformations with an Automated RE-EDS Sampling Procedure

Benjamin Ries, Karl Normak, R. Gregor Weiß, Salomé Rieder, Emília Pécora de Barros, Candide Champion, Gerhard König, Sereina Riniker*

[*] *Laboratory of Physical Chemistry, ETH Zurich, Vladimir-Prelog-Weg 2, 8093 Zurich, Switzerland*
Email: sriniker@ethz.ch

1 Parameter Exploration

A fast transition of the initial maximally contributing end state to the desired maximally contributing end state was observed by monitoring the maximally contributing end state over time. The transition occurred latest after 0.5 ns, and the system remained in the biased end state for the rest of the simulation time. In both water and complex simulations, the desired end state was sampled about 99% of the simulation time with the exception of L19 in water (Table S1). To inspect if the optimized state simulations' results sufficiently represent the target states, a comparison between the target state obtained potential-energy distributions in the EDS simulations with MD simulations consisting of only the target state was conducted (Figure S1).

Table S1: Fraction of the simulation time f_i^{mc} (in %) that the desired end state was sampled as the maximally contributing state during the EDS simulation to optimize the coordinates for a desired end state.

Ligand	Water	Complex
L1	99.84	99.97
L17	99.99	99.97
L19	36.07	99.98
L20	99.99	100
L21	100	99.97

Table S2: Potential thresholds for occurrence sampling (T_i^{phys}) and undersampling (T_i^{us}) determined during the parameter exploration (in kJ mol^{-1}).

Ligand	Water		Complex	
	T^{phys}	T^{us}	T^{phys}	T^{us}
L1	-582.96	-436.05	-737.37	-516.41
L17	-572.41	-419.16	-717.95	-492.83
L19	-579.13	-415.91	-738.95	-483.78
L20	-636.00	-492.75	-759.01	-549.35
L21	-656.22	-488.43	-805.30	-539.78

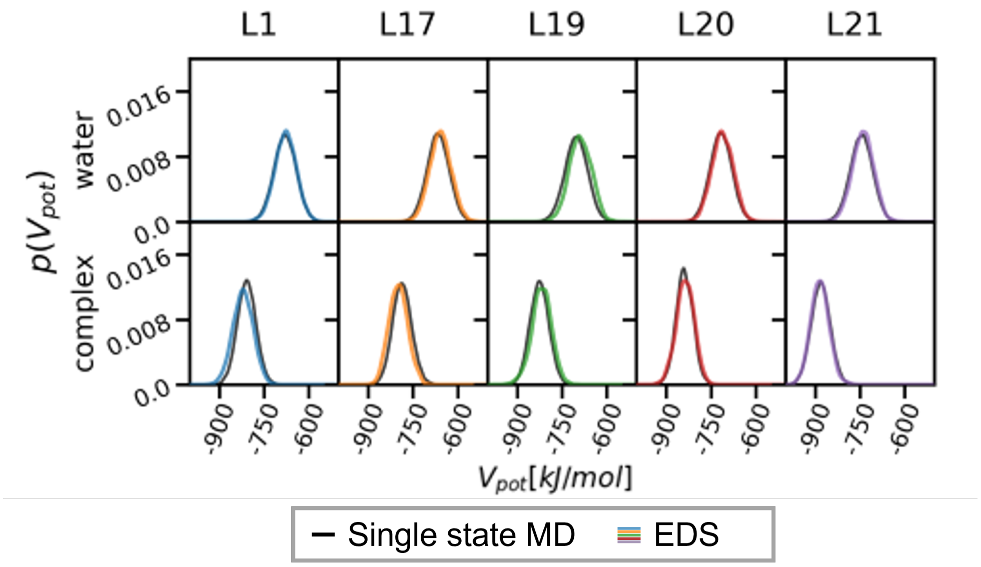

Figure S1: Comparison of the potential-energy distribution obtained from a standard MD simulation of a given end state (black) and from an EDS simulation with the given end state favoured (colored) from the first step of the RE-EDS workflow.

2 Energy Offset Estimation

The relative energy offsets $\Delta\Delta E_{ji}^{R}$ are compared with the experimental relative binding free energies $\Delta\Delta G_{ji}^{\text{bind}}$ in Figure S2. The root mean squared error (RMSE) between $\Delta\Delta E_{ji}^{R}$ obtained with RE-EDS 1SS and $\Delta\Delta G_{ji}^{\text{bind}}$ is 12.6 kJ mol^{-1}. Outliers are mainly related to L19. With the RE-EDS SSM approach, the RMSE was reduced to 7.0 kJ mol^{-1}. No clear outliers were observed in this case. Thus, the use of the SSM approach is recommended for RE-EDS simulations.

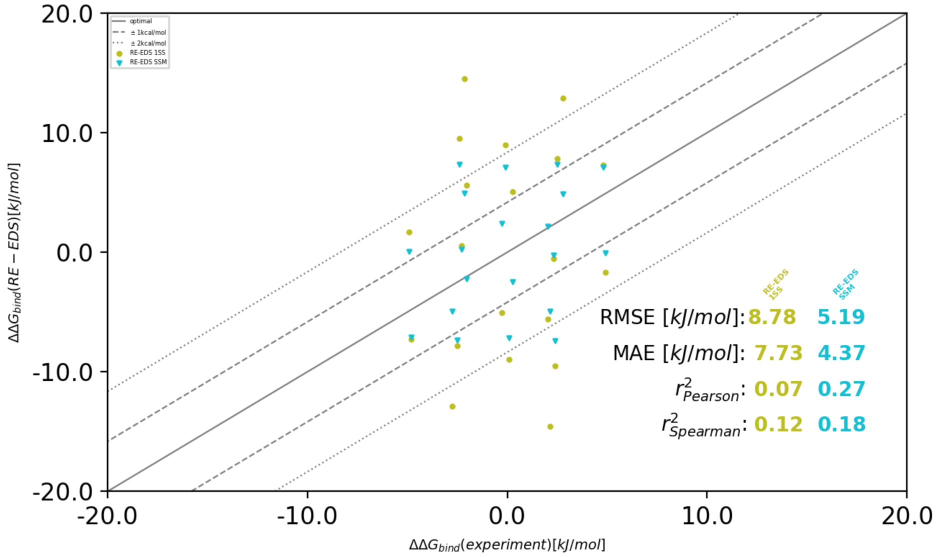

Figure S2: Comparison of the relative energy offsets $\Delta\Delta E_{ji}^{R}$ in water and complex with the experimental relative binding free energies $\Delta\Delta G_{ji}^{\text{bind}}$. The energy offsets were estimated from RE-EDS simulations using the 1SS (green) or SSM (blue) approach to select the starting configurations of the replicas.

3 Optimization of the s-Distribution and the energy offsets

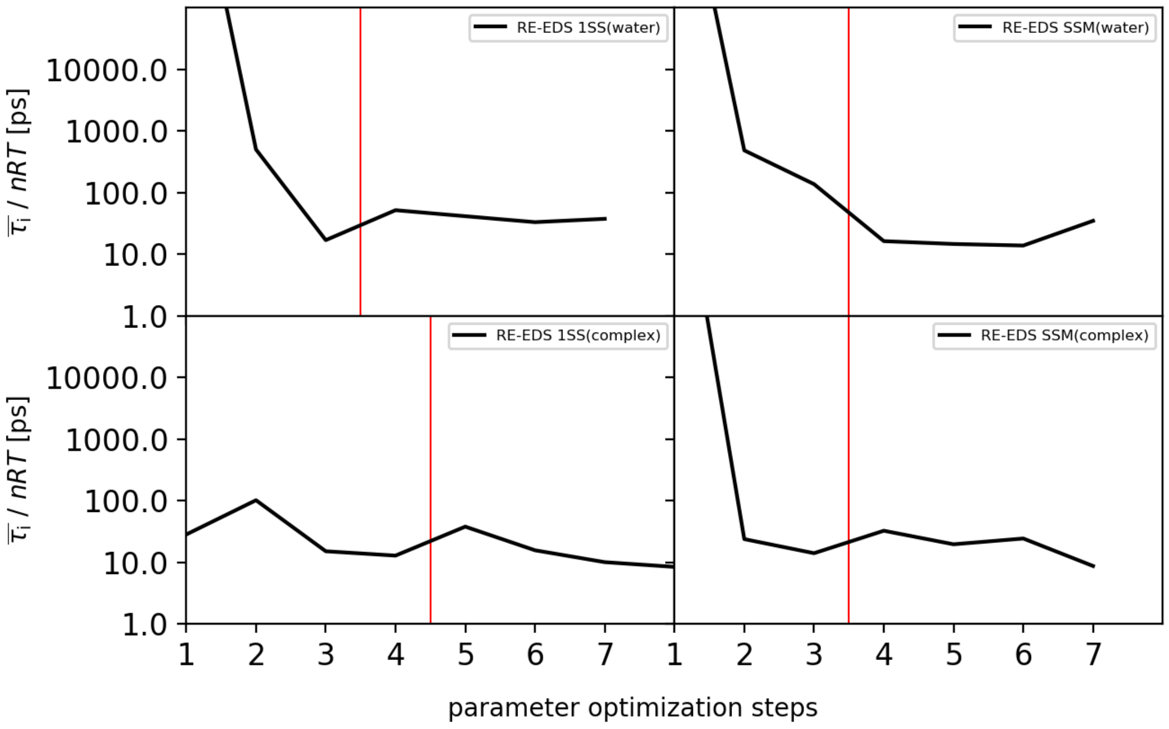

Figure S3: Average round-trip time as a function of the optimization steps i ($\overline{\tau}_i$) on a logarithmic scale. The red line indicates the switch from s-optimization to energy offset rebalancing.

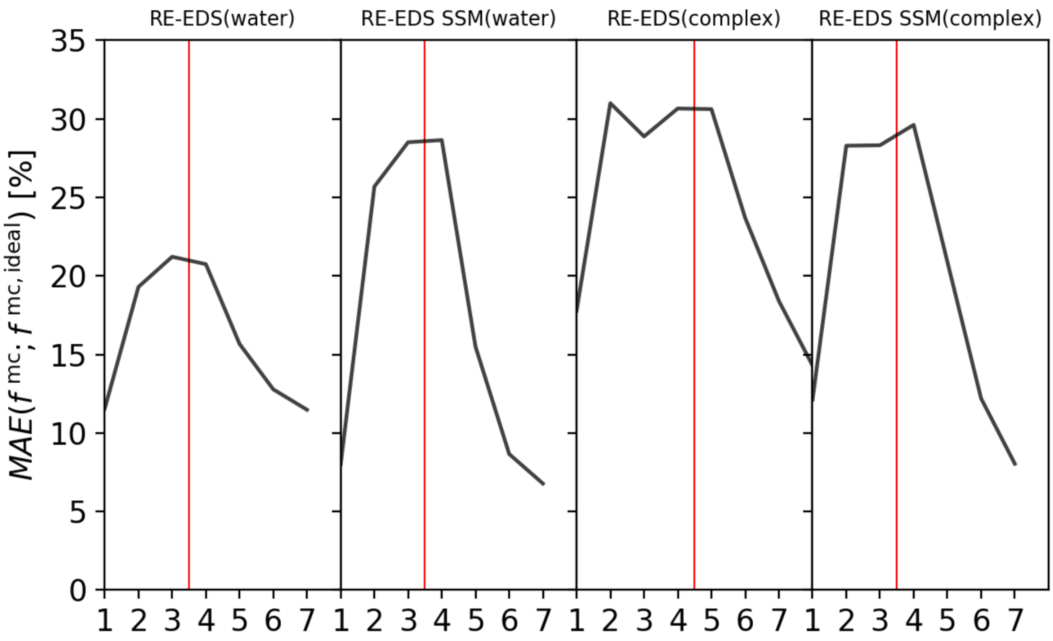

Figure S4: Mean absolute deviation (MAE, in percentage) of the observed state sampling f_i^{mc} from the ideal equal distribution $f_i^{\mathrm{mc,ideal}}$ during the short optimization simulations. The red line indicates the switch from s-optimization to energy offset rebalancing.

4 Free-Energy Calculation

Table S3: Free-energy differences in water and in complex calculated from the production run of 3.5 ns of length with the RE-EDS 1SS and RE-EDS SSM approaches.

Ligand		RE-EDS 1SS		RE-EDS SSM	
J	I	water [kJ mol^{-1}]	complex [kJ mol^{-1}]	water [kJ mol^{-1}]	complex [kJ mol^{-1}]
L17	L1	11.9 ± 0.0	17.0 ± 0.8	12.4 ± 0.5	9.4 ± 1.9
L19	L1	2.7 ± 0.0	5.7 ± 1.0	3.1 ± 0.0	8.0 ± 0.0
L20	L1	-47.8 ± 0.0	-47.6 ± 0.9	-47.7 ± 0.0	-48.1 ± 0.0
L21	L1	-61.7 ± 0.06	-63.1 ± 0.8	-61.7 ± 0.0	-64.8 ± 0.0
L19	L17	-9.2 ± 0.0	-11.3 ± 0.6	-9.3 ± 0.5	-1.4 ± 1.9
L20	L17	-59.6 ± 0.0	-64.5 ± 0.1	-60.1 ± 0.5	-57.6 ± 1.9
L21	L17	-73.6 ± 0.0	-80.1 ± 0.1	-74.1 ± 0.5	-74.3 ± 1.9
L20	L19	-50.5 ± 0.0	-53.2 ± 0.6	-50.7 ± 0.0	-56.2 ± 0.0
L21	L19	-64.4 ± 0.0	-68.8 ± 0.6	-64.7 ± 0.0	-72.9 ± 0.0
L21	L20	-13.9 ± 0.0	-15.5 ± 0.2	-14.0 ± 0.08	-16.7 ± 0.0

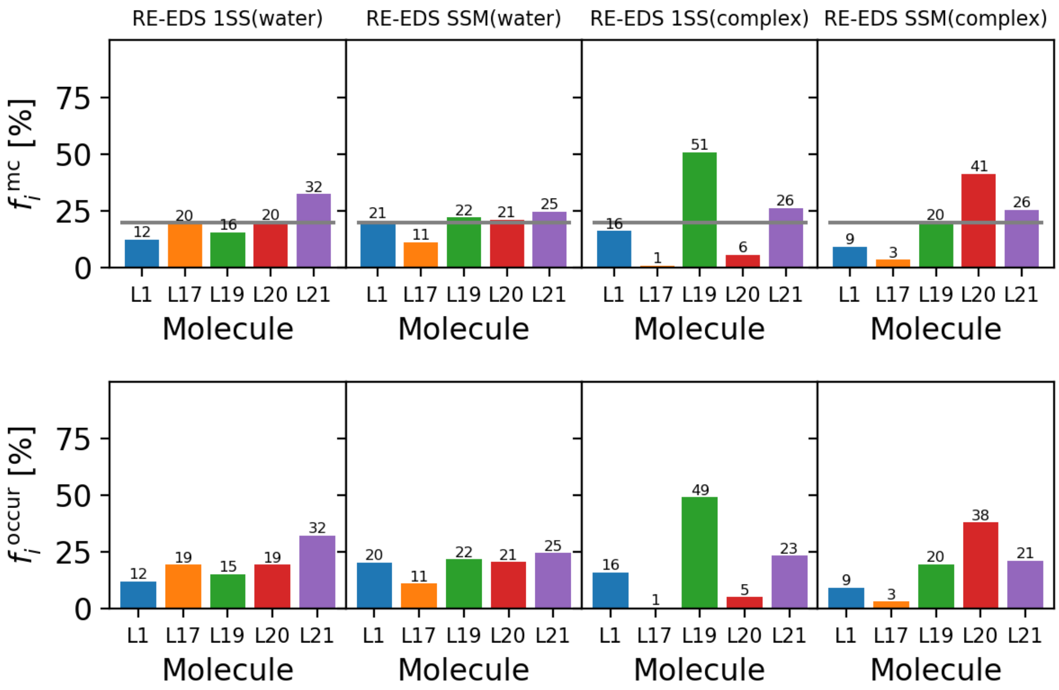

Figure S5: Sampling of the end states in the final production run at replica $s = 1.0$. Sampling was assessed by monitoring the maximally contributing end state (top panels) and by counting all end states a potential energy below T_i^{phys} (see Table S2) (bottom panels). Ideally, the sampling fraction as maximally contributing end state should be $1/N$ (Eq. (8) in the main text) for all end states, indicated as a black horizontal line.

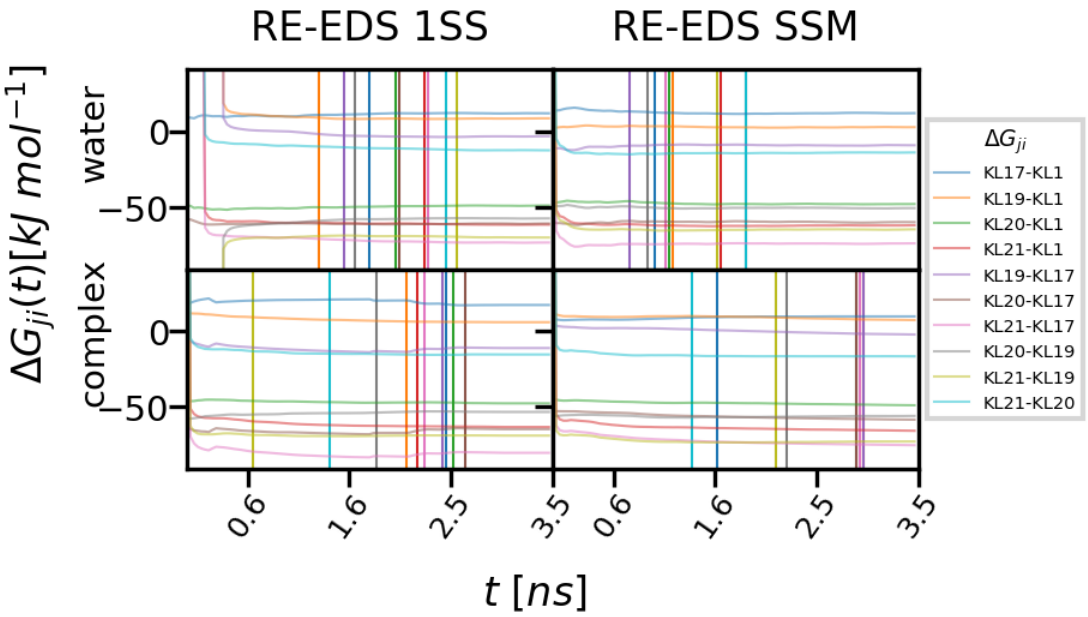

Figure S6: Convergence analysis of the RE-EDS production runs (total 3.5 ns): The free-energy results are plotted as a function of the simulation time. The vertical lines indicate when a particular ΔG_{ji} value was found to be converged (deviation below 1 kJ mol^{-1}).
